# Modern Biology

**Published:** 1893-11-18

**Authors:** 


### Modern Biology.

### III.—ON THE MINUTE STRUCTURE OF CELLS.

In attacking the problems connected with the minute structure of the protoplasm, considered as apart from its more obviously differentiated portions, as, for example, the nucleus, we are brought face to face with the most serious difficulties which arise from the fact that our conclusions are for the most part necessarily founded on a study of the dead tissues instead of the active living cell. Wherein exactly lies the difference between the living and the dead it is impossible definitely to state, but we cannot keep too clearly before us the enormous difference which really does exist between the theoretically smallest possible particle of protoplasm and the numerous nitrogenous and other compounds into which this particle becomes degraded so soon as it has ceased to be alive. The great task which lies before the investigator in this difficult and delicate line of research is that of ensuring the preservation of the architectural configuration of the living protoplasm in the dead substance, in the same way as the configuration of the original printed type remains clearly impressed upon a piece of calcined newspaper, in spite of its otherwise altered nature.

It has always been the aim of good observers to effect this, and since the examination of uninjured protoplasm seldom leads to decisive results, it is constantly necessary to verify and check every conclusion in the most varied manner, whether by the application of staining reagents or of different solvents. These, by possessing the power of discriminating between the various constituents of the cell, enable the investigator to confirm his impressions in detail. But with all the advantages of the best methods in the hands of thoroughly trained microscopists, the difficulties are well-nigh overwhelming; the evidence is at best but fragmentary and often illusory, and he is the best investigator who, while most fertile in interpretation, is most cautious in drawing his conclusions.

What has been said will prepare the reader to anticipate a good deal of disagreement in the results arrived at by the pioneers in this work. Some observers regard protoplasm as consisting of a homogeneous mass, and consider that the minute particles (microsomes) which the microscope reveals to us represent foreign matter—food or protein it may be, but not protoplasm. Others see in them the expression of a definite structure, and, on the whole, the gradually improving methods of research favour this conception. Protoplasm, for these observers, consists of a denser reticulum or meshwork, bathed in a mobile fluid mass, while the "microsomes" are optical phenomena due to the crossing or joining of the delicate threads.

Others, again, with Bütschli, hold a view which is in some ways akin to the reticular theory, namely, that there is present a denser portion which is disposed in much the same way as the walls of a honeycomb, the cavities being filled, as before, with a more fluid substance. Bütschli has devised a most interesting series of experiments in illustration of his views, the more important of which may be mentioned here. He finds that certain emulsions of olive oil with watery solutions of salts, such as potassium carbonate, when placed in water form masses bearing a remarkable resemblance to amœbæ. The emulsion particles execute movements just like those of this animal; a similar streaming of coarser granules is visible in its interior, and what is more striking still, is that there exists a curious similarity in the most minute structure. This, in the frothy emulsion, is that of a honeycomb, and certainly the appearance presented by it, when compared with the protoplasm, such as it may be seen in the hairs of the mallow plant, suggests that an identical structure is present in both. A delicate partitioning can often be made out in both cases, and the "microsomes," which appear in each, can be shown to be due to the fact that three walls always converge to a point, and therefore that they are optical phenomena, and not objective particles. But it must be borne in mind that there is really a vast difference between such a substance as protoplasm, which probably consists of a number of complex bodies, and such a simple mixture as oil and a salt or gelatine solution, whose movements as well as its structure can be demonstrated to be due merely to surface tensions. At the same time, the remarkable similarity is most suggestive, and perhaps indicates the direction along which we may yet successfully seek for a physical explanation of those apparently inexplicable phenomena of movement and structure exhibited by the ultimate protoplasmic particles.

But if the structure of the *cell* protoplasm, which we may conveniently distinguish from that of the nucleus, as cytoplasm, is still involved in obscurity, we do possess a considerable mass of knowledge respecting this nucleus. Here, too, most of the facts which have been placed beyond the reach of doubt have also been reached by the systematic and judicious use of stains and other reagents, for in the living cell the nucleus only looks like a more brightly refractive body, and betrays but little of that extraordinary complexity which really belongs to it.

The action of stains, such as hæmatoxylin, or many of the aniline dyes, demonstrates the existence of a remarkable fibrillar structure which greedily absorbs the colouring matter, whilst the rest of the nucleus remains unstained. The nucleus is delineated from the cell-protoplasm by a pellicle or membrane which, however, under certain circumstances is absent, as will be seen later. Amongst the fibrillar network there may be seen one or more bodies, which all also stain deeply, and which are known as nucleoli. When the structure of the nucleus is investigated more in detail, new features of interest also become evident. It is seen that the substance which absorbs the stain is not diffused evenly through the meshwork, but that it is gathered in isolated discs or droplets, and thus the chromatin (or stainable substance) is distinguishable from the body of the network. These details appear at first sight, perhaps, trifling, but their fundamental significance is grasped when the subject of cellular and nuclear division comes before us, and it may be at once stated that it is probable that in this nuclear fibril, with its contained chromatin, lies the seat of those directive forces which influence the particular form of development which the cell as a whole goes through, and which we class together under the title of heredity.

